# Physical Activity and Breast Cancer Prevention: Possible Role of Immune Mediators

**DOI:** 10.3389/fnut.2020.557997

**Published:** 2020-10-08

**Authors:** Yitong Xu, Connie J. Rogers

**Affiliations:** ^1^Intercollege Graduate Degree Program in Integrative and Biomedical Physiology, Huck Institutes of the Life Sciences, The Pennsylvania State University, University Park, PA, United States; ^2^Department of Nutritional Sciences, The Pennsylvania State University, University Park, PA, United States; ^3^Center for Molecular Immunology and Infectious Disease, Huck Institutes of the Life Sciences, The Pennsylvania State University, University Park, PA, United States; ^4^Penn State Cancer Institute, Hershey, PA, United States

**Keywords:** exercise, immunity, inflammation, recurrence, survival

## Abstract

There is strong evidence that physical activity (PA) reduces risk, recurrence, and mortality from breast cancer. Emerging data suggest that PA induces changes in inflammatory and immune mediators that may contribute to beneficial effects on breast cancer outcomes. Thus, the goal of this review was to evaluate the evidence linking the protective benefit of PA to modulation of immune responses in breast cancer. A literature search was conducted to identify studies that evaluated the impact of PA on tumor and immune outcomes in breast cancer patients and in mammary tumor models. Nineteen studies investigated the effect of PA interventions on cancer immune outcomes using preclinical breast cancer models. Tumor growth was reduced in 11 studies, unchanged in three studies, and increased in one study. Spontaneous metastasis was reduced in two studies and survival was improved in four studies. Frequently assessed immune outcomes include splenic cell number and function, circulating inflammatory cytokines, and intratumoral immune cells and inflammatory markers. Circulating inflammatory cytokine responses were heterogeneous in preclinical models. Within the tumor microenvironment (TME), several studies documented a change in the infiltration of immune cells with an increase in effector cells and a reduction in immune suppressive cells. Twenty-three studies investigated the effect of PA interventions on immune outcomes in breast cancer patients. Thirteen studies used aerobic PA interventions and 10 studies used a combination of aerobic and resistance exercise interventions. Cycling and treadmill activities were the most commonly used PA modalities. Circulating immune cells and inflammatory cytokines were the most frequently assessed immune outcomes in the clinical studies. Among the 19 studies that evaluated a PA intervention during the post treatment period, 10 reported a reduction in the levels of at least one inflammatory cytokine. No inflammatory cytokines were quantified in the three studies that evaluated a PA intervention during treatment with chemotherapy. Immune outcomes within the tumor were assessed in only one study performing a PA intervention prior to surgery. Results from preclinical and clinical studies suggest that PA exerts heterogeneous effects on inflammatory cytokines, but may alter the gene expression profile and immune infiltrates in the tumor which may result in a reduction in immunosuppressive factors. However, additional studies are needed to better understand the effect of PA on immune outcomes in the TME.

## Introduction

Breast cancer is the most commonly diagnosed cancer and leading cause of cancer death among women worldwide (1). Compelling observational data suggest that higher levels of physical activity are associated with reduced risk of pre- and post-menopausal breast cancer (2, 3). Achieving the World Health Organization's recommendation for leisure time physical activity (≥10 MET- or 2.5 hours/week of moderate-intensity activity) is associated with 12% risk reduction for breast cancer (RR = 0.88; 95% CI 0.84–0.91) (4).

Increased physical activity may reduce body mass and/or change body composition, which are also breast cancer risk factors (2). Thus, physical activity and body mass index (BMI) are interrelated, and their independent contribution to breast cancer risk has been difficult to determine. Several studies demonstrate that the protective effect of physical activity on breast cancer risk varies based on BMI status (5–7). However, a number of observational studies demonstrate a beneficial effect of physical activity independent of changes in BMI (8–11). In particular, one prospective cohort study (*n* = 19,196) demonstrates a negative association between post-menopausal breast cancer incidence and physical activity across all BMI categories, and a positive association between post-menopausal breast cancer incidence and BMI across all levels of physical activity, suggesting independent effects of these risk factors (12). In addition, the relationship between physical activity and breast cancer risk reduction appears to be independent of estrogen receptor status, adult weight gain, or postmenopausal hormone therapy (5, 8, 11). Furthermore, physical activity is associated with a significantly delayed onset of breast cancer among BRCA1 and BRCA2 mutation carriers (13).

In addition to a beneficial effect of physical activity on breast cancer risk, physical activity significantly lowers the risk of breast cancer recurrence, breast cancer-specific and all-cause mortality. A recent meta-analysis including over 45,000 breast cancer patients from 32 prospective cohort studies and four randomized controlled trials demonstrates a significantly lower risk of breast cancer recurrence (pooled hazard ratios range from 0.79 to 0.76) and a lower relative risk of all-cause mortality (pooled hazard ratios range from 0.75 to 0.52) among women with higher physical activity levels (14). In addition, a meta-analysis including 22 prospective cohort studies demonstrates that participants who reported high lifetime physical activity had a significantly lower risk of all-cause (HR = 0.82, 95% CI 0.70–0.96) and breast cancer-related death (HR = 0.73, 95% CI 0.54–0.98) compared to the least active women (15). Women with higher recent recreational pre-cancer diagnosis physical activity, post-diagnosis physical activity levels, or meeting the recommended physical activity guidelines post-diagnosis (≥8 MET-hours/week), also had a significantly lower risk of all-cause mortality (HR = 0.73, 95% CI 0.65–0.82; HR = 0.52, 95% CI 0.43–0.64; HR = 0.54, 95% CI 0.38–0.76, respectively) and breast cancer-related mortality (HR = 0.84, 95% CI 0.73–0.97; HR = 0.59, 95% CI 0.45–0.78; HR = 0.67, 95% CI 0.50–0.90, respectively) compared to the least active women (15). Lastly, high compared to low post-diagnosis physical activity is also associated with a reduction in risk of breast cancer-specific mortality in estrogen receptor-positive/progesterone receptor-positive (ER^+^/PR^+^), ER^−^/PR^−^, and triple-negative breast cancer (TNBC) patients (16). Combined, these studies demonstrate that physical activity is beneficial in reducing breast cancer risk and in improving clinical outcomes after a breast cancer diagnosis independent of a variety of host factors (e.g., BMI strata, receptor subtype and presence of genetic risk factors). These results suggest that either there are multiple biological mechanisms underlying the protective effect of physical activity on cancer outcomes and/or the underlying biological mechanism(s) are universal to women of varying genotype or phenotype. Numerous biological mediators of the cancer prevention effects of physical activity have been proposed and include changes in body composition, improvements in metabolic function, a reduction in estrogen availability, and changes in inflammatory and immune mediators (17–19). The metabolic and endocrine pathways altered by physical activity have been evaluated in numerous studies and these results are reviewed elsewhere (17, 18). However, fewer studies have evaluated the potential link between changes in immune and inflammatory mediators and the protective effects of physical activity on breast cancer outcomes, which is the focus of the current review.

The immune system is an important component of endogenous cancer prevention. Cells of the immune system can prevent tumor growth and progression through the recognition and destruction of transformed, tumorigenic cells. Several mechanisms contributing to spontaneous anti-tumor immunity have been characterized in humans and animals models (20, 21). CD8^+^ cytotoxic T cells (CTLs) and natural killer (NK) cells are primarily responsible for killing tumor cells, while CD4^+^ helper T cells can enhance their cytotoxic function by producing cytokines such as IFNγ (21, 22). Immunodeficiency can increase the risk of tumor development, and once established, tumors produce many immunosuppressive factors that can impair anti-tumor immune responses (20, 21). Immunosuppressive cells from both the myeloid and the lymphoid lineages are recruited in response to tumor-derived signals to facilitate immune escape, including myeloid-derived suppressor cells (MDSCs) and regulatory T cells (Tregs), respectively (21, 22). In addition, a wide variety of cytokines, chemokines and other immune modulatory molecules also play important roles in dictating the immune response systemically and in the tumor microenvironment (TME) (21, 22). In healthy animals and humans, chronic, aerobic physical activity enhances T cell and NK cell function and vaccine responses (23–25) and also reduces infection rates, chronic inflammation and age-related immunosenescence (26–30). However, less is known about the effect of physical activity on anti-tumor immunity. Emerging evidence suggests that physical activity may result in an increase in effector cell number and function, a reduction in immunosuppressive cells, and an altered cytokine profile in animal models of breast cancer (31–35). However, a definitive link between physical activity and improved cancer immunity is yet to be established.

To date, several reviews have summarized the effect of various types of physical activity (resistance, aerobic or the combination) on immune outcomes in patients of any cancer type (36, 37), and in breast cancer patients across the cancer continuum (38–40). However, no studies have comprehensively reviewed the evidence in animal models and breast cancer patients linking physical activity and immune modulation. Therefore, the objective of the current review was to evaluate and summarize both the preclinical and clinical evidence relating the protective benefit of chronic, aerobic physical activity on breast cancer outcomes to the modulation of immune responses, in both peripheral tissues/circulation and the TME.

## Methods

### Search Strategy

A systematic literature search was conducted using PubMed (Medline), Web of Science and CENTRAL to identify studies that evaluate the effect of physical activity (PA) interventions on tumor and immune outcomes in preclinical mammary tumor models and breast cancer patients. The search strategy included three components: (1) PA-related terms; (2) molecular and cellular components of the immune system and immunological processes important in cancer immunity; (3) breast cancer-related terms (complete search strategy provided in Supplementary Table 1). Supplemental hand searching was also conducted to identify additional studies from the reference lists of relevant review and primary research articles.

### Inclusion and Exclusion Criteria

Only primary research articles written in the English language were included. This review aimed to evaluate the effect of chronic, aerobic PA interventions (such as running and swimming) in the context of breast cancer. Therefore, all observational studies and studies conducted in non-cancer animal or human subjects were excluded. Studies performing resistance exercise only, conditioning or stretching exercise (e.g., Tai Chi or yoga), or a single bout of exercise were also excluded. Because clinical studies frequently performed a combination of aerobic and resistance exercise interventions to prevent muscle wasting and cachexia in cancer patients, these studies were included. Preclinical and clinical studies that performed solely resistance exercise were excluded. Eligible preclinical studies must have reported the effect of PA interventions on tumor outcomes (tumor incidence, growth, metastasis, or survival). Clinical studies rarely investigated the effect of PA interventions on tumor outcomes, thus were not restricted by this criterion. Studies were included only if they assessed the effect of PA on immune outcomes, including immune cell number and/or function, cytokines, and other immune and inflammatory markers. Studies that used multiple interventions (such as those investigating the effect of PA and dietary supplements given alone and in combination) were included, as long as it was possible to compare groups receiving no intervention to PA intervention alone.

### Study Selection and Quality Assessment

After de-duplication of the search results, a title/abstract screening was first performed on all records to identify potentially eligible studies, followed by a full-text screening to determine the eligibility of each study based on the inclusion and exclusion criteria. For preclinical studies, quality assessment was performed according to the SYstematic Review Center for Laboratory animal Experimentation's (SYRCLE's) risk of bias tool (41) (Supplementary Table 2). For clinical studies, quality assessment was performed according to National Institutes of Health Quality Assessment Tool for Controlled Intervention Studies, and for Before-After (Pre–Post) Studies With No Control Group (42) (Supplementary Table 3).

### Data Extraction and Analysis

The following information was extracted from each eligible study for analysis: bibliographic information (author and publication year), animal model or human subject characteristics, PA intervention details, tumor outcomes and immune outcomes. Due to the heterogeneity in study design and immune outcomes assessed, data were synthesized in narrative form and a meta-analysis was not performed. However, a Preferred Reporting Items for Systematic Reviews and Meta-Analyses (PRISMA) flow diagram was created to describe the study selection process (43) (Supplementary Figure 1). Preclinical and clinical studies were summarized separately and were classified based on the timing of PA interventions in relation to tumor initiation or cancer treatment, respectively. For clinical studies, comparisons of immune outcomes between breast cancer patients performing PA intervention vs. sedentary control were recorded where possible. In studies using a single-group design where all participants performed PA interventions, comparisons between baseline and post-intervention values were recorded.

## Results

Details regarding the study selection process are outlined in a PRISMA flow diagram (Supplementary Figure 1). In brief, 6,812 records were identified after duplicates were removed, and 6,577 were excluded following title and abstract screening. Of the 235 remaining studies, 42 met inclusion criteria and were included in the review. The 42 eligible studies included 19 preclinical and 23 clinical studies investigating the effect of PA interventions on immune outcomes in breast cancer. Results from preclinical and clinical studies were summarized separately below.

### Preclinical Findings

Nineteen studies investigated the effect of PA interventions on cancer immune outcomes using preclinical breast cancer models (Table 1). Transplantable tumor models were used in 14 studies (31, 32, 34, 35, 44–50, 53–55), among which six used the 4T1 (or its subclone 4T1.2) TNBC murine mammary tumor model (32, 34, 35, 44, 48, 54). Carcinogen-induced (*n* = 3) (33, 56, 57) and genetically-engineered/spontaneous (*n* = 2) (51, 52) mammary tumor models were also used. Treadmill running (*n* = 11) was the most commonly used PA modality, followed by voluntary wheel running (*n* = 5), swimming (*n* = 3), and motorized wheel running (*n* = 2). Of these, two studies compared the effect of different PA modalities on tumor and immune outcomes (33, 46).

**Table 1:** Preclinical findings on the effects of PA on immune outcomes in breast cancer.

**References**	**Tumor model**	**PA modality**	**PA protocol**	**PA/tumor timeline**	**Tumor outcomes**	**Immune outcomes**	
						**PA effect**	**PA no effect**
**1. PA intervention prior to tumor initiation (*****n*** **= 2)**							
Hagar et al. (32)	Female Balb/c mice, 4T1 mammary tumor (orthotopic inoculation)	Motorized wheel running	4 m/min, 10 min/day to 10 m/min, 26 min/day, 5 days/wk	PA lasted 8 wks until 72h before tumor inoculation	↓Mean tumor size and tumor doubling time; ↑Survival	↑Circulating leukocytes, neutrophils, monocytes; ↓Intratumoral Tregs, CD8^+^/FoxP3^+^ ratio	Intratumoral CD8^+^ T cells
Wang et al. (44)	Female Balb/c mice, 4T1 mammary tumor (orthotopic inoculation)	Treadmill running	15 m/min, 60 min/day	PA lasted 20 days until tumor inoculation	↓Tumor volume and weight	↑Serum IL-6; ↑Splenic and tumor-infiltrating NK cells	–
**2. PA intervention from pre to post tumor initiation (*****n*** **= 10)**							
Shalamzari et al. (45)	Female BALB/c mice, MC4-L2 mammary tumor (s.c. inoculation)	Treadmill running	14 m/min, 25 min/day to 20 m/min, 40 min/day, 5 days/wk	PA for 8 wks before, 6 wks after, or from 8 wks before to 6 wks after tumor inoculation	↓Tumor growth	↓IL-6 within the tumor (EX from pre to post, or post tumor inoculation)	–
Turbitt et al. (34)	Female BALB/c mice, 4T1.2 mammary tumor (orthotopic inoculation)	Voluntary wheel running (alone or in combination with 10% energy restriction)	Access to running wheels	PA from 8 wks before to 5 wks after tumor inoculation	↓Tumor growth; ↓Lung and femur metastasis; ↑Survival (all effects by PA+ER)	↓MDSC accumulation, ↑CD4^+^ T cell proliferation in the spleen (PA+ER); ↓Tumor-infiltrating MDSCs, ↑CD8^+^ T cells, CD8^+^:MDSC ratio (PA+ER); Altered inflammatory and immune pathways within the tumor	Number of splenic Tregs
Hoffman-Goetz et al. (46)	Female BALB/c mice, MMT 66 mammary tumor (i.v. inoculation)	Treadmill vs. voluntary wheel running	18 m/min, 30 min/day (treadmill) or access to running wheels	PA for 8 wks before, 3 wks after, or from 8 wks before to 3 wks after tumor inoculation	No change in experimental lung metastasis	↑Splenic NKCC, LAK activity (magnitude varied by EX intervention)	–
Bacurau et al. (31)	Male Wistar rats, Walker 256 mammary tumor (s.c. inoculation)	Treadmill running (high-intensity)	30 min/day at 85% VO_2_max, 5 days/wk	PA from 8 wks before to 15 days after tumor inoculation	↓Tumor mass/body weight ratio; ↑Survival	↑Plasma IL-1, TNFα; ↑Mesenteric LN lymphocyte proliferation	Plasma IL-2; Peritoneal macrophage phagocytosis
Bacurau et al. (47)	Male Wistar rats, Walker 256 mammary tumor (s.c. inoculation)	Treadmill running (moderate intensity)	1 h/day at 60% VO_2_max, 5 days/wk	PA from 8 wks before to 14 days after tumor inoculation	↑Survival	↑Mesenteric LN lymphocyte proliferation; ↑Peritoneal macrophage phagocytosis	–
Molanouri et al. (48)	Female Balb/c mice, 4T1 mammary tumor (s.c. inoculation)	Treadmill running	50 min/day at 50–70% VO_2_max, 5 days/wk	PA from 6 wks before to 6 wks after tumor inoculation	No change in tumor volume	↓IL-4 within the tumor; ↓IL-4, ↑IFNγ, ↑IFNγ/IL-4 ratio produced by stimulated splenocytes	TNFα, IL-6, IFNγ within the tumor
Almeida et al. (49)	Male Swiss mice, Ehrlich mammary tumor (s.c. inoculation)	Swimming (moderate vs. high intensity)	1 h/day at 50 or 80% of maximal capacity, 5 days/wk	PA from 4 wks before to 2 wks after tumor inoculation	↓Tumor volume and weight (moderate intensity)	↓Macrophage and neutrophil infiltration in the tumor (moderate intensity)	–
Woods et al. (50)	C3H/HeN mice, SCA-1 mammary tumor (s.c. inoculation)	Treadmill running (moderate vs. exhaustive)	18 m/min, 5% grade, 30 min/day or increasing speed until exhaustion (2–3 h)/day	PA from 3 days before to 14 days after tumor inoculation	No change in tumor incidence or progression	↑Tumor-infiltrating macrophage phagocytic activity (moderate EX)	–
Murphy et al. (51)	Female C3(1)SV40Tag mice, spontaneous mammary tumor	Treadmill running	20 m/min, 5% grade, 60 min/day at 70% VO_2_max, 6 days/wk	PA initiated at 4 wks of age and lasted 20 wks	↓Spontaneous tumor number; ↓Tumor volume	↓Plasma MCP-1 and IL-6	–
Goh et al. (52)	Female PyMT transgenic mice, spontaneous mammary tumor	Voluntary wheel running	Access to running wheels	PA initiated at 42 days of age and lasted 10 wks	↓Tumor growth	↓CCL22, ↑CXCR4 gene expression within the tumor	Spleen weight
**3. PA intervention post tumor initiation (*****n*** **= 7)**							
Khori et al. (53)	Female BALB/c mice, MC4-L2 mammary tumor (s.c. inoculation)	Treadmill running	16–18 m/min, 10–14 min/day, 5 days/wk	PA initiated at tumor inoculation and lasted 5 wks	↓Tumor size	↓IL-6 within the tumor	–
Bianco et al. (54)	Female Balb/c mice, 4T1 mammary tumor (orthotopic inoculation)	Swimming	15–45 min/day, 5 days/wk	PA initiated at tumor inoculation and lasted 4 wks	↓Tumor volume	↑Th1 markers, ↓Th2 markers, ↓%Treg cells in the spleen; ↓%TIDCs, ↑CD80^+^/CD86^+^ TIDCs; ↑%BMDCs, CD80^+^/CD86^+^ BMDCs, ↓IL-10 production by BMDCs	%Tumor-infiltrating CD4^+^ and CD8^+^ T cells, macrophages
Wennerberg et al. (35)	Female Balb/c mice, 4T1 mammary tumor (s.c. inoculation)	Treadmill running	18 m/min, 30 min/day, 5 days/wk	PA initiated 8 days after tumor inoculation and lasted 3 wks	↓Tumor growth; No change in lung metastasis	↓MDSCs, ↑Ki-67^+^ and CD69^+^ NK cells in the spleen; ↓Tumor-infiltrating MDSCs, ↑tumor-infiltrating CD8^+^/CD69^+^ T cells	Total T cells or NK cells in the spleen; Tumor-infiltrating CD8^+^/Ki-67^+^ T cells
Buss and Dachs (55)	Female C57BL/6 *ApoE*^−/−^ mice, EO771 mammary tumor (orthotopic inoculation)	Voluntary wheel running	Access to running wheels	PA initiated at tumor inoculation and lasted an average of 16 days	↓Incidence of abdominal metastasis; No change in tumor growth rate	–	Number of tumor-infiltrating T cells, %CTLs or %Tregs of total T cells; Serum MCP-1
Faustino-Rocha et al. (56)	Female Sprague-Dawley rats, MNU-induced mammary tumor	Treadmill running	20 m/min, 60 min/day, 5 days/wk	PA initiated after tumor induction and lasted 35 wks	↓Tumor number; No change in tumor weight	–	Serum IL-6, CRP
Thompson et al. (33)	Female Sprague-Dawley rats, MNU-induced mammary tumor	Motorized vs. voluntary wheel running	Access to motorized (constant speed at 40 m/min) or non-motorized wheels	PA initiated 1 wk after tumor induction	↓Tumor incidence and multiplicity	↓Plasma IL-1α, IL-1β, TNFα; ↑Plasma IL-2, GM-CSF, IFNγ, IL-4, IL-6, IL-10	Plasma CRP
Saez et al. (57)	Female Sprague-Dawley rats, DMBA-induced mammary tumor	Swimming	30 min/day, 5 days/wk	PA initiated after the appearance of the 1st tumor and lasted 1–2 months	↑Tumor growth; No change in tumor multiplicity	–	% Circulating NK cells

*Studies were ranked by the total length of PA intervention (high to low) in each category. ↑, increase (compared to control group); ↓, decrease (compared to control group); IFN, interferon; IL, interleukin; TNF, tumor necrosis factor; CRP, C-reactive protein; MCP, monocyte chemoattractant protein; GM-CSF, granulocyte-macrophage colony-stimulating factor; CCL, CC chemokine ligand; CXCR, CXC chemokine receptor; BMDC, bone marrow-derived dendritic cell; TIDC, tumor-infiltrating dendritic cell; NK, natural killer; NKCC, NK cell cytotoxicity; LAK, lymphokine activated killer; CTL, cytotoxic T lymphocyte; Th, T helper; Treg, regulatory T cell; MDSC, myeloid-derived suppressor cell; LN, lymph node; VO_2_max, maximum oxygen consumption rate; DMBA, 7,12-dimethylbenz(a)anthracene; MNU, N-methyl-N-nitrosourea; s.c., subcutaneous; i.v. intravenous; EX, exercise; ER, energy restriction*.

Included studies were highly heterogeneous in the frequency, duration and intensity of the PA intervention, as well as in the length and timeline of the PA intervention period. To evaluate if PA performed before or after tumor initiation may result in different tumor and immune outcomes, we classified the included studies into three categories: PA intervention prior to (*n* = 2) (32, 44), from pre to post (*n* = 10, including the spontaneous tumor models) (31, 34, 45–52), and post tumor initiation (*n* = 7) (33, 35, 53–57). Despite the heterogeneity in study designs, most studies reported positive effects of PA on tumor outcomes. Tumor incidence and/or multiplicity was reduced in three studies (33, 51, 56) and unchanged in two studies (50, 57). Tumor growth was reduced in 11 studies (31, 32, 34, 35, 44, 45, 49, 51–54), unchanged in three studies (48, 50, 55), and increased in one study (57). Spontaneous metastasis was reduced in two studies (34, 55) and unchanged in one study (35), and one additional study reported no change in experimental lung metastasis (46). Survival was improved in all four studies reporting cancer survival outcomes (31, 32, 34, 47).

Frequently assessed immune outcomes include splenic cell number and function, circulating inflammatory cytokines, and intratumoral immune cells and inflammatory markers. Among splenic effector cells, one study reported no change in the percentage of total T or NK cells, but an increase in proliferating (Ki-67^+^) and activated (CD69^+^) NK cells (35). Three other studies reported an increase in the number of NK cells (44) and a polarization of Th1/Th2 balance toward Th1 response (48, 54), respectively. Increased splenic CD4^+^ T cell proliferation [by PA plus energy restriction (ER)] (34), NK cell cytotoxicity (48) and lymphokine activated killer (LAK) activity (46) were each reported in one study. Among splenic immunosuppressive cells, one study reported a reduction in the number of MDSCs and no change in the number of Tregs (by PA+ER) (34), and two other studies reported a reduction in either the percentage or number of MDSCs (35) and Tregs (54), respectively.

Circulating inflammatory cytokine responses were heterogeneous in preclinical models. TNFα was reduced in one study (33) and increased in another study (31). IL-1(β) was reduced in one study (33) and increased in another study (31). MCP-1 was reduced in one study (51) and unchanged in another study (55). IL-6 was reduced (51), increased (33), or unchanged (56) in three individual studies. CRP was unchanged in two studies (33, 56). Fewer studies have evaluated circulating cytokines related to effector functions. Of those, IL-2 was increased in one study (33) and unchanged in another study (31), and IFNγ was increased in one study (33).

Within the TME, two studies reported an increase in the percentage of infiltrating CD4^+^ and CD8^+^ T cells (34) or activated (CD8^+^/CD69^+^) T cells (35), respectively. One study reported an increase in the percentage of tumor-infiltrating NK cells (44). Yet three studies reported no change in the percentage or number of CD4^+^ or CD8^+^ T cells (32, 54, 55). In addition, two studies assessed immune outcomes in the non-tumor-draining (mesenteric) lymph nodes and found an increase in lymphocyte proliferation (31, 47). Among the immune suppressive cells, the percentage of MDSCs was reduced in two studies (34, 35), and Treg number or *Foxp3* gene expression were reduced in two studies (32, 34). Additionally, two studies reported a reduction in the percentage or number of tumor-infiltrating macrophages (49, 54), while a third study reported an increase in macrophage phagocytic activity (50). Intratumoral inflammatory cytokines were also assessed in several studies. One study reported no change in TNFα, IL-6, or IFNγ within the tumor (48), while three other studies reported a reduction in IL-6 (45, 53) or *Ifng* gene expression (34), respectively. Of note, one study evaluated the gene expression profile within the TME (34), and found global downregulation of genes important in metastasis, immune checkpoint molecules, and chemokines associated with MDSC and Treg recruitment (by PA alone or in combination with ER).

### Clinical Findings

Twenty-three studies investigated the effect of PA interventions on immune outcomes in breast cancer patients (Table 2). Thirteen studies used solely aerobic PA interventions and 10 studies used a combination of aerobic and resistance exercise interventions. Cycling and treadmill activities were the most commonly used aerobic PA modalities. Similar to preclinical studies, the frequency, duration, intensity and timeline of the PA interventions were highly heterogeneous in the clinical studies. To evaluate if the timing of the PA intervention in relation to cancer treatment may significantly impact immune outcomes, we classified the included studies into three categories: PA intervention performed prior to (*n* = 1) (58), during (*n* = 3) (39, 59, 60), and post treatment (*n* = 19) (61–79).

**Table 2:** Clinical findings on the effects of PA on immune outcomes in breast cancer.

						**Immune outcomes**	
**References**	**Participants**	**PA/treatment timeline**	**Intervention groups**	**PA modality**	**PA protocol**	**PA effect**	**PA no effect**
**1. PA intervention prior to treatment (*****n*** **= 1)**							
Ligibel et al. (58)	Newly diagnosed breast cancer patients	Between enrollment and surgery	Intervention (*n* = 24) Control (*n* = 20)	Aerobic + resistance exercise	180 min aerobic + 40 min strength training/wk, for 29.3 days (on average)	↑Inflammatory and immune pathways within the tumor; ↓Tumor-infiltrating Tregs	Serum CRP, IL-6; tumor-infiltrating CD4^+^, CD8^+^, CD56^+^, CD163^+^ cells
**2. PA intervention during treatment (*****n*** **= 3)**							
Mijwel et al. (59)	Breast cancer patients	During adjuvant chemotherapy	Intervention (*n* = 60) Control (*n* = 57)	Aerobic exercise (cycling)	20 min aerobic exercise (at RPE 13–15) + 10 min HIIT (at RPE 16-18)/session, 2 sessions/wk, 16 wks	–	Circulating lymphocyte and neutrophil concentration
Schmidt et al. (39)	Breast cancer patients	During adjuvant chemotherapy	Intervention (*n* = 20) Control (*n* = 26)	Aerobic exercise (cycling)	45 min/session at Borg level of 11-14, 2 sessions/wk, 12 wks	–	Circulating CD3, CD4, CD8, αβ, γσ T cells, CD19 B cells, CD16/CD56 NK cells
Kim et al. (60)	Breast cancer patients	During chemotherapy	Pre–post intervention (*n* = 20)	Aerobic exercise (walking)	40–60 min/session at 40–60% HRR, 5 sessions/wk, 12 wks	–	Circulating leukocytes, lymphocytes, helper or cytotoxic T cells, NK cells, NKT cells
**3. PA intervention post treatment (*****n*** **= 19)**							
Giallauria et al. (61)	Breast cancer patients	Within 5 years after mastectomy or conservative surgery	Intervention (*n* = 61) Control (*n* = 33)	Aerobic exercise (treadmill activity or cycling)	30 min/session at 70% VO_2_max, 3 sessions/wk for 3 months + 1 session/wk for 9 months; 12 months in total	↓Serum HMGB-1	Serum hsCRP, IL-6
Sturgeon et al. (62)	*BRCA1/2^+^* breast cancer patients	≥4 months after breast cancer treatment	Intervention (*n* = 19) Control (*n* = 16)	Aerobic + resistance exercise	3 days/wk aerobic + 3 days/wk resistance exercise, 160 min/wk in total, 12 months	↓Serum IL-6	Serum IL-1β, IL-8, TNFα
Peters et al. (63)	Breast cancer patients	≥6 months after surgery	Pre–post intervention (*n* = 24)	Aerobic exercise (cycling)	5 times/wk for 5 wks + 2–3 times/wk for 6 months; 7 months in total	↑NKCC	Number or percentage of circulating NK cells
Peters et al. (64)	Breast cancer patients	≥6 months after surgery	Pre–post intervention (*n* = 24)	Aerobic exercise (cycling)	30–40 min/day, 5 times/wk for 5 wks + 2–3 times/wk for 6 months; 7 months in total	↑%Circulating granulocytes, ↓lymphocytes and monocytes; ↑Monocyte phagocytosis	–
Loo et al. (65)	Breast cancer patients	Within 6-60 months post treatment	Pre–post intervention (*n* = 8)	Aerobic exercise (Hula Dance)	Supervised (60 min/session, 2 sessions/wk) + home-based dance (15 min/session, 3 sessions/wk), 6 months	↓Serum IL-1β, IL-2, IL-4, IL-5, IL-10, GM-CSF, IFNγ, TNFα (at 12 but not 6 months)	Serum CRP, IL-6, IL-8
Dethlefsen et al. (66)	Breast cancer patients	Post primary treatment	Intervention (*n* = 37) Control (*n* = 37)	Aerobic (cycling) + resistance exercise plus exercise counseling	Supervised exercise 90 min/session, 1 session/wk, 6 months	↓Serum TNFα	Serum IL-6, IL-8, IL-10
Hutnick et al. (67)	Breast cancer patients	≥2 wks after the completion of chemotherapy	Intervention (*n* = 21) Control (*n* = 15)	Aerobic (treadmill running, walking) + resistance exercise	20 min aerobic (at 60–75% functional capacity) + resistance training/session, 3 sessions/wk, 3–6 months	↑%Circulating CD4^+^/CD69^+^ cells; ↑Mitogen-stimulated lymphocyte proliferation	Circulating CD3^+^, CD4^+^, CD8^+^ T cells, B cells, NK cells^a^; IFNγ, IL-6 production by mitogen-stimulated lymphocytes; Plasma IFNγ, IL-6, sIL-6R, sgp130, IFNγ/IL-6 ratio
Dieli-Conwright et al. (68)	Post-menopausal breast cancer patients (BMI ≥ 30 kg/m^2^)	After the completion of radiotherapy and/or chemotherapy	Intervention (*n* = 10) Control (*n* = *n* = 10)	Aerobic + resistance exercise	150 min moderate-vigorous (at 65-80% HRmax) aerobic exercise + 2–3 days of resistance exercise training/wk, 16 wks	↓Plasma CRP, IL-6, IL-8; ↓%M1 and ↑%M2 macrophages, ↑IL-12 in the adipose tissue	–
Fairey et al. (69)	Post-menopausal breast cancer patients	After the completion of surgery, radiotherapy, and/or chemotherapy	Intervention (*n* = 24) Control (*n* = 28)	Aerobic exercise (cycling)	15–35 min/session at 70–75% VO_2_max, 3 sessions/wk, 15 wks	↑NKCC; ↑Spontaneous lymphocyte proliferation	Mitogen-stimulated lymphocyte proliferation; Blood mononuclear cell phenotypes and cytokine production, neutrophil function^a^
Fairey et al. (70)	Post-menopausal breast cancer patients	After the completion of surgery, radiotherapy, and/or chemotherapy	Intervention (*n* = 24) Control (*n* = 28)	Aerobic exercise (cycling)	15–35 min/session at 70–75% VO_2_max, 3 sessions/wk, 15 wks	↓Serum CRP	–
Rogers et al. (71)	Breast cancer patients	Post primary treatment	Intervention (*n* = 11) Control (*n* = 9)	Aerobic + resistance exercise	150 min moderate-intensity aerobic + 2 sessions of resistance training/wk, gradually shifted to home-based exercise, 3 months	–	Serum IL-6, IL-8, IL-10, TNFα, IL-6/IL-10 ratio, IL-8/IL-10 ratio, TNFα/IL-10 ratio
Rogers et al. (72)	Post-menopausal breast cancer patients	≥4 wks after final primary treatment administration	Intervention (*n* = 20) Control (*n* = 22)	Aerobic (walking) + resistance exercise	Aerobic (40 min/session at 48-52% HRR, 4 sessions/wk) + resistance (2 sessions/wk) exercise, 3 months	↓Serum IL-10	Serum IL-6, IL-8, TNFα, IL-6/IL-10 ratio, IL-8/IL-10 ratio, TNFα/IL-10 ratio
Alizadeh et al. (73)	Non-metastatic and hormone-responsive breast cancer patients	≥1 month after the completion of radiotherapy and/or chemotherapy	Intervention (*n* = 24) Control (*n* = 24)	Aerobic exercise (treadmill walking)	38 min/session, at 50–95% HRmax, 3 days/wk, 12 wks	↑Serum TNFα, ↓IL-6, TNFα/IL-10 and IL-6/IL-10 ratio; ↑IL-4 production by mitogen-stimulated PBMCs	Serum IL-10, IL-1β; IFNγ production by mitogen-stimulated PBMCs
Gomez et al. (74)	Post-menopausal breast cancer patients	2–5 years post treatment	Intervention (*n* = 8) Control (*n* = 8)	Aerobic (cycling) + resistance exercise	20–30 min aerobic (at 70–80% HRmax) + resistance training/session, 3 sessions/wk, 8 wks	Prevent ↑in serum CTACK; ↓Serum IL-15, MIF, IL-10/TNFα ratio	Various serum cytokines
Jones et al. (75)	Post-menopausal breast cancer patients	≥6 months after completion of adjuvant treatment	Intervention (*n* = 36) Control (*n* = 32)	Aerobic exercise (primarily walking)	30 min/session at 60–80% HRmax, 5 sessions/wk, 8 wks	–	Serum IL-6, CRP, TNFα
Nieman et al. (76)	Breast cancer patients	Within 4 years after surgery, radiotherapy, and/or chemotherapy	Intervention (*n* = 6) Control (*n* = 6)	Aerobic (walking) + resistance exercise	30 min aerobic (at 75% HRmax) + resistance training/session, 3 sessions/wk, 8 wks	–	Number of circulating lymphocytes, neutrophils, T cells, NK cells; NKCC
Tizdast et al. (77)	Breast cancer patients (BMI > 25 kg/m^2^)	≥6 months after the completion of surgery, radiotherapy, and/or chemotherapy	Continuous EX (*n* = 9) Interval EX (*n* = 8) Control (*n* = 6)	Aerobic exercise (treadmill activity)	Continuous (15–40 min at 30–50% THR) or interval (5 × 3 to 8 × 5 min at 40–60% THR) exercise, 3 times/wk, 8 wks	–	Serum IL-6, TNFα, hCRP
Karimi et al. (78)	Breast cancer patients (BMI > 25 kg/m^2^)	Post radiotherapy and/or chemotherapy	Intervention (*n* = 10) Control (*n* = 10)	Aerobic exercise (water-based)	20–60 min/session at 50–75% HRR, 4 sessions/wk, 6 wks	↓Serum IL-10, hsCRP	–
Zimmer et al. (79)	Breast cancer patients	During stationary rehabilitation	Pre–post intervention (*n* = 60)	Personalized exercise recommendations	9–15 MET/wk for 3 wks, plus 1-week stay at the clinic 4 and 8 months later	↓Serum CRP	Serum TNFα, IL-6, MIF

*Studies were ranked by the total length of PA intervention (high to low) in each category. Sample sizes with immune outcomes were recorded. ^a^Data not shown. ↑, increase (compared to control group or pre-intervention level); ↓, decrease (compared to control group or pre-intervention level); BMI, body mass index; HIIT, high-intensity interval training; EX, exercise; VO_2_max, maximum oxygen consumption rate; RPE, rate of perceived exertion; HRR, heart rate reserve; HRmax, maximum heart rate; THR, target heart rate; MET, metabolic equivalent of task; NK, natural killer; NKCC, NK cell cytotoxicity; NKT cell, natural killer T cell; IFN, interferon; IL, interleukin; TNF, tumor necrosis factor; CRP, C-reactive protein; hsCRP, high-sensitivity CRP; hCRP, human CRP; GM-CSF, granulocyte-macrophage colony-stimulating factor; MIF, macrophage migration inhibiting factor; HMGB-1, high mobility group box-1 protein; CTACK, cutaneous T cell-attracting chemokine; PBMC, peripheral blood mononuclear cell*.

Circulating immune cells and inflammatory cytokines were the most frequently assessed immune outcomes in the clinical studies. In the 19 studies that evaluated a PA intervention during the post treatment period, 10 reported a reduction in at least one inflammatory cytokine including IL-6, IL-8, CRP, MIF, TNFα, and HMGB-1 (61, 62, 65, 66, 68, 70, 73, 74, 78, 79). However, no study measured all of these inflammatory mediators and some studies found no effect of the PA intervention on one or more of these cytokines. Two studies found an increase in NK cell cytotoxicity (63, 69) and mitogen-induced lymphocyte proliferation (67, 69) and one study reported an increase in monocyte phagocytosis (64). Four studies quantified immune cell populations in circulation (63, 64, 67, 76), but only one reported an increase in the percentage of CD4^+^/CD69^+^ cells (67).

No inflammatory cytokines were quantified in the three studies that evaluated a PA intervention during treatment with chemotherapy. Circulating immune cell populations including T cells, B cells, NK cells, total lymphocytes, neutrophils and monocytes were measured in all three studies and PA had no effect on the distribution of immune cells.

Immune outcomes within the tumor were assessed in only one study performing a PA intervention prior to treatment (58). In this study, one month of PA intervention prior to surgery resulted in the upregulation of multiple immune and inflammatory pathways within the TME, including cytokine-cytokine receptor interactions, NK-mediated cytotoxicity, T-cell receptor signaling, antigen processing and presentation. No change was found in tumor-infiltrating CD4^+^ or CD8^+^ T cells or macrophages, while a trend toward a reduction in tumor-infiltrating Tregs was observed.

## Discussion

Gaining a better understanding of the biological mediators underlying the beneficial effect of physical activity on breast cancer outcomes is needed in order to make clearer recommendations regarding the dose, duration, and frequency of physical activity needed to achieve the beneficial effects observed to date, and to make evidence-based decisions about possible synergies between physical activity prescriptions and standard and emerging therapeutic strategies. Numerous studies have explored the biological mechanisms linking physical activity and improved breast cancer outcomes, including those related to inflammatory and immune responses. Thus, the goal of this review was to summarize the results from studies that have examined physical activity interventions and immune outcomes in animal models and women with breast cancer, in order to make conclusions regarding the role of physical activity on immune modulation in breast cancer.

Strong epidemiological evidence suggests that physical activity is associated with reduced breast cancer risk (2–4). Consistent with data collected in observational studies, 13 out of the 15 preclinical studies that assessed primary tumor growth demonstrated that physical activity reduces mammary tumor growth or volume. This physical activity-induced reduction in tumor growth occurred regardless of whether the physical activity interventions occurred prior to, from pre to post, and only post tumor initiation. Tumor incidence and/or multiplicity were most commonly assessed in spontaneous and carcinogen-induced breast cancer models. Among these studies, tumor incidence was reduced in one study (33), while tumor multiplicity was reduced in three studies (33, 51, 56) and unchanged in another study (57). In the two studies that utilized PA interventions of the shortest duration (16–17 days), no change in tumor incidence and/or growth was observed (50, 55). These data suggest that longer exposure to regular bouts of physical activity may be needed to achieve a beneficial effect on tumor outcomes.

Strong evidence from observational and randomized controlled trials also suggests that physical activity is associated with reduced breast cancer recurrence and cancer-specific and all-cause mortality (14–16). Metastasis contributes significantly to the mortality of breast cancer patients (80). Events in the metastatic pathway include the release of cancer cells from the primary tumor; invasion of surrounding tissue including stroma, lymphatics, and/or blood vessels (as circulating tumor cells, or CTCs); and colonization of target organs as disseminated tumor cells (DTCs) (81). DTCs may undergo cell death via recognition by immune effector cells; initiate rapid cell proliferation; or enter a state of tumor dormancy. Dormant cells have been proposed to either exist as single cells in cell cycle arrest (82), or to be actively dividing, but with restricted proliferation due to either lack of angiogenesis (83) or immune surveillance (84, 85). Metastatic dormancy is widespread in breast cancer, although the molecular basis is not completely known. Metastatic burden was evaluated in four preclinical studies where physical activity was performed from pre to post, or post tumor initiation. Two studies found a reduction in spontaneous metastasis to the lung, femur or abdominal cavity (34, 55), while two others found no change in spontaneous or experimental lung metastasis, respectively (35, 46). In addition, four studies reported an improvement in survival in physically active animals (PA alone or in combination with ER) (31, 32, 34, 47). In all four studies, mice or rats were exposed to eight weeks of a PA intervention prior to tumor inoculation, but were removed from the PA intervention at varying intervals post tumor inoculation (e.g., at the time of, or two or five weeks after tumor inoculation). These results suggest that PA prior to tumor development, at least in the mammary tumor models studied to date, may be important in reducing metastatic burden and preventing tumor-related death. Thus, it is plausible that physical activity may be reducing recurrence and increasing survival in breast cancer patients by suppressing metastases to distant sites. However, additional studies are needed to confirm these findings and to explore the biological mechanisms contributing to a PA-induced reduction in metastases and mortality in mammary tumor models.

Both preclinical and clinical studies that have explored the effect of PA on cancer immune outcomes report highly variable results. A wide range of immunological parameters has been assessed, yet each parameter is only assessed in a small number of studies with mixed results. Due to limitations in the accessibility of human samples, peripheral blood is often the only tissue compartment from which immune cells or inflammatory mediators are assessed in clinical studies. Over half (53%) of the studies evaluating a PA intervention in the post treatment period reported a reduction in at least one inflammatory cytokine (IL-6, IL-8, CRP, MIF, TNFα, and HMGB-1) in plasma or serum (61, 62, 65, 66, 68, 70, 73, 74, 78, 79). However, no study measured all of these inflammatory mediators in the same patients and some studies found no effect of the PA intervention on one or more of these cytokines within the same study. Preclinical studies that evaluated circulating inflammatory markers reported mixed results. This heterogeneity in the response of inflammatory mediators in preclinical models may be attributed to differences in the timing of sample collection with respect to the last bout of activity, the use of different mammary tumor models that may have different cytokine profiles, and/or the presence of growing tumors that can be a direct source of inflammatory cytokines. Cytokines and chemokines play a critical role in cancer-related inflammation, regulating both host and malignant cells in the tumor microenvironment (86). Furthermore, metastasis is driven by inflammatory signals and the infiltration of inflammatory cells into the primary tumor (87). PA can reduce inflammatory mediators associated with obesity and other chronic diseases (88, 89) in the absence of tumor. Thus, evaluating the cytokine milieu in response to physical activity interventions in both preclinical models and in breast cancer patients is an important outcome to pursue in future studies to determine if changes in inflammatory mediators correlate with clinical outcomes.

In addition to circulating factors, preclinical studies often assess immune outcomes in the spleen and tumor microenvironment. Emerging evidence suggests that PA may induce beneficial changes in splenic immunity, represented by an increase in T cell proliferation and Th1 cytokine production (34, 48, 54), NK cell number and cytotoxicity (44, 46), and a reduction in the immunosuppressive populations induced by tumors including MDSCs and Tregs (34, 35, 54). Within the TME, there is a similar trend of increased effector cells (CD4^+^ and CD8^+^ T cells and NK cells) and a reduction in pro-tumor immune suppressors (MDSCs and Tregs) (32, 34, 35, 44) in animals exposed to PA interventions. In a study using the 4T1.2 murine mammary tumor model (34), PA alone or in combination with ER induced global changes in the gene expression profile within the TME, featured by a downregulation of chemokines important in MDSC and Treg recruitment (CCL5, CCL20, CCL22), immune checkpoint (programmed cell death protein 1, PD-1) and other inhibitory molecules (indoleamine 2,3-dioxygenase, IDO). Consistent with this, a clinical study performing a pre-surgical PA intervention (58) reported an upregulation in multiple pathways within the TME, including those involved in effector cell function and inflammatory signaling. These findings suggest that PA may be preventing the escalation of immunosuppression while promoting anti-tumor immune response within the TME, which may improve responses to both chemotherapy and immunotherapy.

Overall, it has been hypothesized that the modulation of the immune system is a candidate for the development of more effective therapies in breast cancer. However, breast cancer is a heterogeneous disease, with HER2^+^ and TNBC being more immunogenic compared to hormone receptor (HR)-positive subtypes (90–92). HER2^+^ and TNBC are more likely to be infiltrated by tumor-infiltrating lymphocytes (TILs) and to express programmed death ligand-1 (PD-L1) in the TME than ER- or PR-expressing luminal tumors (93–95). Moreover, the quantity of TILs is a prognostic indicator of disease recurrence-free survival and overall survival, especially for TNBC (96–98). Higher TIL concentration is also predictive of response to neoadjuvant chemotherapy in all subtypes of breast cancer (99). The clinical success of immune checkpoint inhibitors (ICI) in several other cancer types has established immunotherapy as a fundamental pillar of cancer treatment (100). However, recent clinical trials in breast cancer immunotherapy utilizing ICIs have yielded mixed results (90, 101). Initial phase I/II trials using PD-1/PD-L1 inhibitors as single-agent therapy report an overall response rate (ORR) under 10% in unselected populations (102–104). However, higher ORRs (12–24%) are observed in several trials in which enriched subgroups of TNBC patients were involved, including PD-L1^+^ patients with high TIL level, and when the PD-1/PD-L1 inhibitor was administered as an earlier line of therapy (90, 103, 105). In an effort to improve response rates and overcome acquired resistance, various combination strategies have been developed to explore the potential synergy between ICI and radiation, chemotherapy, targeted therapy, and other immunotherapies (90, 101, 106). This combinatorial approach has led to improved clinical outcomes and FDA approval for the use of a PD-L1 inhibitor (atezolizumab) in combination with paclitaxel protein-bound for patients with unresectable locally advanced or metastatic TNBC whose tumors express PD-L1 (107). Given the potential for physical activity to modulate both systemic immunity and immune infiltration in the TME, physical activity may be a possible strategy to use in combination with chemotherapy and emerging immunotherapies. To date, no clinical trials have explored the effect of physical activity on immunotherapy outcomes. However, a preclinical study using the 4T1 murine breast cancer model demonstrates that PA enhances the anti-tumor effect of a combination of PD-1 blockade and focal radiotherapy (35). The improved therapeutic response was associated with reduced PD-1 expression by splenic CD8^+^ T cells, splenic and tumor-infiltrating NK cells, and a reduction in tumor-infiltrating MDSCs. Despite the limited amount of evidence, emerging data suggest that PA may be altering the TME, which could enhance the efficacy of immunotherapies. However, additional studies are needed to explore the use of ICI alone or in combination with other standard therapies, with and without physical activity, to determine if any additive or synergistic effects are observed.

The current review has several limitations. There is much heterogeneity in the prescription of PA interventions (modality, frequency, duration, intensity, and timing), as well as the preclinical cancer models used and characteristics of human participants. Additionally, many of the clinical studies are exploratory trials with immune outcomes assessed in a small sample size. Thus, limited definitive conclusions can be made on the potential effect of PA on cancer immune outcomes.

**Figure 1:**
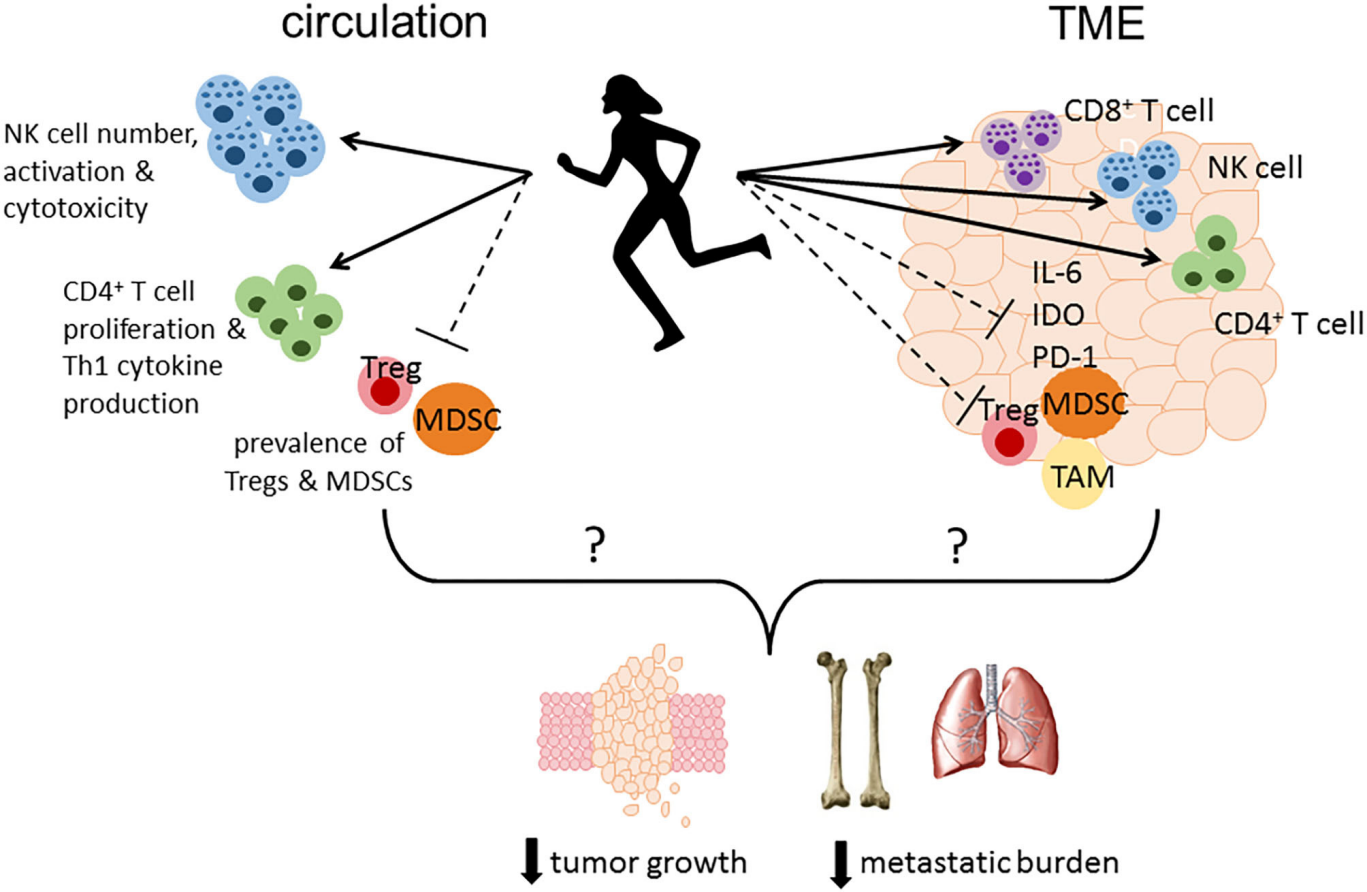
Proposed immune mechanisms altered by physical activity that may play a role in reducing tumor growth, metastatic burden and mortality. Physical activity may modulate immune responses in circulation resulting in enhanced immune surveillance by increasing the number, activation status, and cytotoxicity of NK cells, enhancing CD4^+^ T cell proliferation and Th1 cytokine production, and reducing the number or percentage of immunosuppressive MDSCs and Tregs. Furthermore, physical activity may alter the immune landscape within the TME leading to greater infiltration of effector cells (CD4^+^ and CD8^+^ T cells, NK cells) concurrent with a reduction in immunosuppressive factors including IL-6, PD-1, and IDO, and less accumulation of MDSCs, TAMs, and Tregs. TME, tumor microenvironment; IL-6, interleukin-6; PD-1, programmed cell death protein 1; IDO, indoleamine 2,3-dioxygenase; MDSC, myeloid-derived suppressor cell; TAM, tumor-associated macrophage; Treg, regulatory T cell.

In summary, existing preclinical studies consistently demonstrate a beneficial effect of PA on breast cancer outcomes, with the most abundant evidence on reducing primary mammary tumor growth. Emerging preclinical evidence also suggests a potential role of PA in reducing tumor incidence, metastasis and improving survival. However, exploration on the potential immunological mechanisms underlying such beneficial effect is still in its early phase. Findings from clinical studies suggest that physical activity may have an impact on circulating inflammatory cytokines. However, additional studies are warranted to better understand which cytokines might be impacted by activity and if these changes correlate with clinical outcomes. Physical activity may modulate immune responses in circulation resulting in enhanced immune surveillance, and/or alter the immune landscape within the TME leading to greater infiltration of effector cells and a reduction in immune suppression (Figure 1). Additional mechanistic studies are needed to determine the potential immune mediators contributing to physical activity-induced improvement in tumor outcomes, particularly within the TME, and if a causal relationship exists. Well-designed clinical trials are also warranted to confirm if immunological mechanisms underlie the cancer prevention effect of physical activity, as well as to evaluate if physical activity interventions may be used in combination with standard and emerging immunotherapies to improve clinical outcomes in breast cancer patients.

## Author Contributions

CR and YX participated in the conception and design of the work, collection and assembly of data, data analysis and interpretation, and manuscript writing. All authors approved the final version for submission.

## Conflict of Interest

The authors declare that the research was conducted in the absence of any commercial or financial relationships that could be construed as a potential conflict of interest.
