# Supplementary material for: Physical Activity and Breast Cancer Prevention: Possible Role of Immune Mediators
Source: Front Nutr. 2020 Oct 8;7:557997. doi: 10.3389/fnut.2020.557997 (PMC7578403; doi:10.3389/fnut.2020.557997)
Supplement: Supplementary file 1 [file Table_1.docx]

# Supplementary Table 1. Search strategy.

**1.1 PubMed**

("exercise"[mesh] OR "exercise"[tiab] OR "exercises"[tiab] OR "physical activity"[tiab] OR "physical activities"[tiab] OR "physical fitness"[mesh] OR "physical fitness"[tiab] OR "aerobic activity"[tiab] OR "aerobic activities"[tiab] OR "aerobic training"[tiab] OR "endurance training"[tiab] OR "physical endurance"[tiab] OR "physical conditioning"[tiab] OR "walk"[tiab] OR "walking"[tiab] OR "swim"[tiab] OR "swimming"[tiab] OR "running"[tiab] OR "jog"[tiab] OR "jogging"[tiab] OR "cardiovascular activity"[tiab] OR "cardiovascular activities"[tiab] OR "cardiometabolic"[tiab])

AND

("immune"[tiab] OR "immunity"[mesh] OR "immunity"[tiab] OR "immune system"[mesh] OR "immune system phenomena"[mesh] OR "immunotherapy"[mesh] OR "immunotherapy"[tiab] OR "immunotherapies"[tiab] OR "leukocyte"[tiab] OR "leukocytes"[tiab] OR "lymphocyte"[tiab] OR "lymphocytes"[tiab] OR "T cell"[tiab] OR "T cells"[tiab] OR "B cell"[tiab] OR "B cells"[tiab] OR "natural killer"[tiab] OR "NK cell"[tiab] OR "NK cells"[tiab] OR "monocyte"[tiab] OR "monocytes"[tiab] OR "macrophage"[tiab] OR "macrophages"[tiab] OR "dendritic cell"[tiab] OR "dendritic cells"[tiab] OR "neutrophil"[tiab] OR "neutrophils"[tiab] OR "myeloid-derived suppressor cells"[mesh] OR "myeloid-derived suppressor cell"[tiab] OR "myeloid-derived suppressor cells"[tiab] OR "myeloid derived suppressor cell"[tiab] OR "myeloid derived suppressor cells"[tiab] OR "regulatory T cell"[tiab] OR "regulatory T cells"[tiab] OR "Treg"[tiab] OR "Tregs"[tiab] OR "immunosuppression"[tiab] OR "antigen presentation"[tiab] OR "cytokine"[tiab] OR "cytokines"[tiab] OR "chemokine"[tiab] OR "chemokines"[tiab] OR "antibodies"[mesh] OR "antibody"[tiab] OR "antibodies"[tiab] OR "immunoglobulins"[mesh] OR "immunoglobulin"[tiab] OR "immunoglobulins"[tiab] OR "inflammation"[mesh] OR "inflammation"[tiab] OR "inflammations"[tiab] OR "inflammatory"[tiab])

AND

("neoplasms"[mesh] OR "neoplasms"[tiab] OR "neoplasm"[tiab] OR "neoplasia"[tiab] OR "neoplasias"[tiab] OR "cancer"[tiab] OR "cancers"[tiab] OR "tumor"[tiab] OR "tumors"[tiab] OR "tumour"[tiab] OR "tumours"[tiab] OR "malignancy"[tiab] OR "malignancies"[tiab])

NOT review[pt]

AND English[lang]

**1.2 Web of Science**

TOPIC: ("exercise" OR "exercises" OR "physical activity" OR "physical activities" OR "physical fitness" OR "aerobic activity" OR "aerobic activities" OR "aerobic training" OR "endurance training" OR "physical endurance" OR "physical conditioning" OR "walk" OR "walking" OR "swim" OR "swimming" OR "running" OR "jog" OR "jogging" OR "cardiovascular activity" OR "cardiovascular activities" OR "cardiometabolic")

AND

TOPIC: ("immune" OR "immunity" OR "immunotherapy" OR "immunotherapies" OR "leukocyte" OR "leukocytes" OR "lymphocyte" OR "lymphocytes" OR "T cell" OR "T cells" OR "B cell" OR "B cells" OR "natural killer" OR "NK cell" OR "NK cells" OR "monocyte" OR "monocytes" OR "macrophage" OR "macrophages" OR "dendritic cell" OR "dendritic cells" OR "neutrophil" OR "neutrophils" OR "myeloid-derived suppressor cell" OR "myeloid-derived suppressor cells" OR "myeloid derived suppressor cell" OR "myeloid derived suppressor cells" OR "regulatory T cell" OR "regulatory T cells" OR "Treg" OR "Tregs" OR "immunosuppression" OR "antigen presentation" OR "cytokine" OR "cytokines" OR "chemokine" OR "chemokines" OR "antibody" OR "antibodies" OR "immunoglobulin" OR "immunoglobulins" OR "inflammation" OR "inflammations" OR "inflammatory")

AND

TOPIC: ("neoplasm" OR "neoplasms" OR "neoplasia" OR "neoplasias" OR "cancer" OR "cancers" OR "tumor" OR "tumors" OR "tumour" OR "tumours" OR "malignancy" OR "malignancies")

AND

LANGUAGE: English

NOT

DOCUMENT TYPES: Review

**1.3 CENTRAL**

ti.ab.kw.: ("exercise" OR "physical activity" OR "aerobic activity" OR "aerobic training" OR "endurance training")

AND

ti.ab.kw.: ("immune" OR "immunity" OR "immunotherapy" OR "leukocyte" OR "lymphocyte" OR "T cell" OR "B cell" OR "natural killer" OR "NK cell" OR "monocyte" OR "macrophage" OR "dendritic cell" OR "neutrophil" OR "myeloid-derived suppressor cell" OR "cytokine" OR "chemokine" OR "antibody" OR "immunoglobulin" OR "inflammation" OR "inflammations" OR "inflammatory")

AND

ti.ab.kw.: ("neoplasm" OR "neoplasia" OR "cancer" OR "tumor" OR "malignancy")
