# Supplementary material for: Physical Activity and Breast Cancer Prevention: Possible Role of Immune Mediators
Source: Front Nutr. 2020 Oct 8;7:557997. doi: 10.3389/fnut.2020.557997 (PMC7578403; doi:10.3389/fnut.2020.557997)
Supplement: Supplementary file 2 [file Table_2.docx]

# Supplementary Table 2. Quality assessment of preclinical studies according to SYRCLE’s RoB tool.

| **Reference** | **#1** | **#2** | **#3** | **#4** | **#5** | **#6** | **#7** | **#8** | **#9** | **#10** | **Score** |
| --- | --- | --- | --- | --- | --- | --- | --- | --- | --- | --- | --- |
| Hagar 2019 | Yes | Yes | Yes | Yes | No | Unclear | Unclear | Yes | Yes | Yes | 7 |
| Wang 2020 | Yes | Yes | Yes | Yes | No | Unclear | Unclear | Yes | Yes | Yes | 7 |
| Shalamzari 2014 | Yes | Yes | Yes | Yes | No | Unclear | Yes | Yes | Yes | Yes | 8 |
| Turbitt 2019 | Yes | Yes | Yes | Yes | No | Unclear | Yes | Yes | Yes | Yes | 8 |
| Hoffman-Goetz 1994 | Yes | Yes | Yes | Yes | No | Unclear | Yes | Yes | Yes | Yes | 8 |
| Bacurau 2007 | Yes | Yes | Yes | Yes | No | Unclear | Yes | Yes | Yes | Yes | 8 |
| Bacurau 2000 | Unclear | Unclear | Unclear | Yes | No | Unclear | Yes | Yes | Yes | Yes | 5 |
| Molanouri 2019 | Yes | Yes | Yes | Yes | No | Unclear | Yes | Yes | Yes | Yes | 8 |
| Almeida 2009 | Unclear | Unclear | Unclear | Yes | No | Unclear | Unclear | Yes | Yes | Yes | 4 |
| Woods 1994 | Yes | Yes | Yes | Yes | No | Unclear | Yes | Yes | Yes | Yes | 8 |
| Murphy 2011 | Yes | Yes | Yes | Yes | No | Unclear | Yes | Yes | Yes | Yes | 8 |
| Goh 2013 | Yes | Yes | Yes | Yes | No | Unclear | Yes | Yes | Yes | Yes | 8 |
| Khori 2015 | Unclear | Unclear | Unclear | Yes | No | Unclear | Yes | Yes | Yes | Yes | 5 |
| Bianco 2017 | Unclear | Unclear | Unclear | Yes | No | Unclear | Yes | Yes | Yes | Yes | 5 |
| Wennerberg 2020 | Yes | Yes | Yes | Yes | No | Unclear | Yes | Yes | Yes | Yes | 8 |
| Buss and Dachs 2018 | Yes | Yes | Yes | Yes | No | Unclear | Unclear | Yes | Yes | Yes | 7 |
| Faustino-Rocha 2017 | Yes | Yes | Yes | Yes | No | Unclear | Unclear | Yes | Yes | Yes | 7 |
| Thompson 2010 | Yes | Yes | Yes | Yes | No | Unclear | Yes | Yes | Yes | Yes | 8 |
| Saez 2007 | Unclear | Unclear | Unclear | Yes | No | Unclear | Yes | Yes | Yes | Yes | 5 |

Yes: low risk of bias; No: high risk of bias; Unclear: insufficient details to properly assess the risk of bias. Scores of 0-3, 4-7 and 8-10 represent high, moderate, and low risks, respectively.
