# Supplementary material for: Physical Activity and Breast Cancer Prevention: Possible Role of Immune Mediators
Source: Front Nutr. 2020 Oct 8;7:557997. doi: 10.3389/fnut.2020.557997 (PMC7578403; doi:10.3389/fnut.2020.557997)
Supplement: Supplementary file 3 [file Table_3.docx]

**Supplementary Table 3A. Quality assessment of clinical controlled intervention studies according to NIH Quality Assessment Tool.**

| **Reference** | **#1** | **#2** | **#3** | **#4** | **#5** | **#6** | **#7** | **#8** | **#9*** | **#10** | **#11** | **#12** | **#13** | **#14** | **Score** |
| --- | --- | --- | --- | --- | --- | --- | --- | --- | --- | --- | --- | --- | --- | --- | --- |
| Ligibel 2019 | Yes | Yes | Yes | NA | Yes | Yes | Yes | Yes | Yes | Yes | Yes | Yes | Yes | Yes | 13 |
| Mijwel 2019 | Yes | Yes | Yes | NA | No | Yes | Yes | Yes | Yes | Yes | Yes | Yes | Yes | Yes | 12 |
| Schmidt 2018 | Yes | Yes | Yes | NA | NR | Yes | Yes | No | NR | Yes | Yes | No | Yes | No | 8 |
| Giallauria 2014 | Yes | Yes | Yes | NA | Yes | Yes | Yes | Yes | No | Yes | Yes | Yes | Yes | No | 11 |
| Sturgeon 2018 | Yes | Yes | Yes | NA | Yes | Yes | Yes | No | Yes | Yes | Yes | No | Yes | NR | 10 |
| Dethlefsen 2016 | Yes | Yes | Yes | NA | Yes | Yes | CD | CD | Yes | No | Yes | Yes | Yes | Yes | 10 |
| Hutnick 2005 | No | No | No | NA | NR | Yes | CD | CD | Yes | Yes | Yes | No | Yes | Yes | 6 |
| Dieli-Conwright 2018 | Yes | Yes | Yes | NA | NR | Yes | Yes | Yes | Yes | Yes | Yes | No | Yes | NR | 10 |
| Fairey 2005 | Yes | Yes | Yes | NA | Yes | Yes | Yes | Yes | Yes | Yes | Yes | Yes | Yes | Yes | 13 |
| Fairey 2005 | Yes | Yes | Yes | NA | Yes | Yes | Yes | Yes | Yes | Yes | Yes | Yes | Yes | Yes | 13 |
| Rogers 2013 | Yes | Yes | Yes | NA | Yes | Yes | No | Yes | Yes | Yes | Yes | No | Yes | Yes | 11 |
| Rogers 2014 | Yes | Yes | Yes | NA | Yes | Yes | Yes | Yes | Yes | Yes | Yes | No | Yes | Yes | 12 |
| Alizadeh 2019 | Yes | Yes | Yes | NA | Yes | Yes | Yes | Yes | Yes | Yes | Yes | Yes | Yes | NR | 12 |
| Gomez 2011 | Yes | Yes | Yes | NA | Yes | Yes | Yes | Yes | Yes | Yes | Yes | No | Yes | Yes | 12 |
| Jones 2013 | Yes | Yes | Yes | NA | Yes | Yes | Yes | Yes | No | Yes | Yes | No | Yes | Yes | 11 |
| Nieman 1995 | Yes | Yes | NR | NA | NR | Yes | No | Yes | Yes | Yes | Yes | No | Yes | Yes | 9 |
| Tizdast 2016 | Yes | Yes | NR | NA | NR | Yes | Yes | No | NR | Yes | Yes | No | Yes | NR | 7 |
| Karimi 2015 | Yes | Yes | NR | NA | NR | Yes | CD | CD | NR | Yes | Yes | NR | Yes | NR | 6 |

CD, cannot determine; NA, not applicable; NR, not reported. *Adherence of 66% (two-thirds) or higher is considered as high adherence. Scores of 0-4, 5-9 and 10-13 represent high, moderate, and low risks, respectively (13 applicable entries in total).

**Supplementary Table 3B. Quality assessment of clinical before-after (pre-post) studies with no control group according to NIH Quality Assessment Tool.**

| **Reference** | **#1** | **#2** | **#3** | **#4** | **#5** | **#6** | **#7** | **#8** | **#9** | **#10** | **#11** | **#12** | **Score** |
| --- | --- | --- | --- | --- | --- | --- | --- | --- | --- | --- | --- | --- | --- |
| Kim 2015 | Yes | Yes | Yes | NR | NR | Yes | Yes | NA | NR | Yes | No | NA | 6 |
| Peters 1994 | Yes | Yes | Yes | NR | NR | Yes | Yes | NA | NR | Yes | No | NA | 6 |
| Peters 1995 | Yes | Yes | Yes | NR | NR | Yes | Yes | NA | NR | Yes | No | NA | 6 |
| Loo 2019 | Yes | Yes | Yes | No | NR | Yes | Yes | NA | No | Yes | No | NA | 6 |
| Zimmer 2018 | Yes | Yes | Yes | NR | NR | Yes | Yes | NA | NR | Yes | No | NA | 6 |

CD, cannot determine; NA, not applicable; NR, not reported. Scores of 0-3, 4-7 and 8-10 represent high, moderate, and low risks, respectively (10 applicable entries in total).
