# Supplementary figures and images for: Physical Activity and Breast Cancer Prevention: Possible Role of Immune Mediators
Source: Front Nutr. 2020 Oct 8;7:557997. doi: 10.3389/fnut.2020.557997 (PMC7578403; doi:10.3389/fnut.2020.557997)

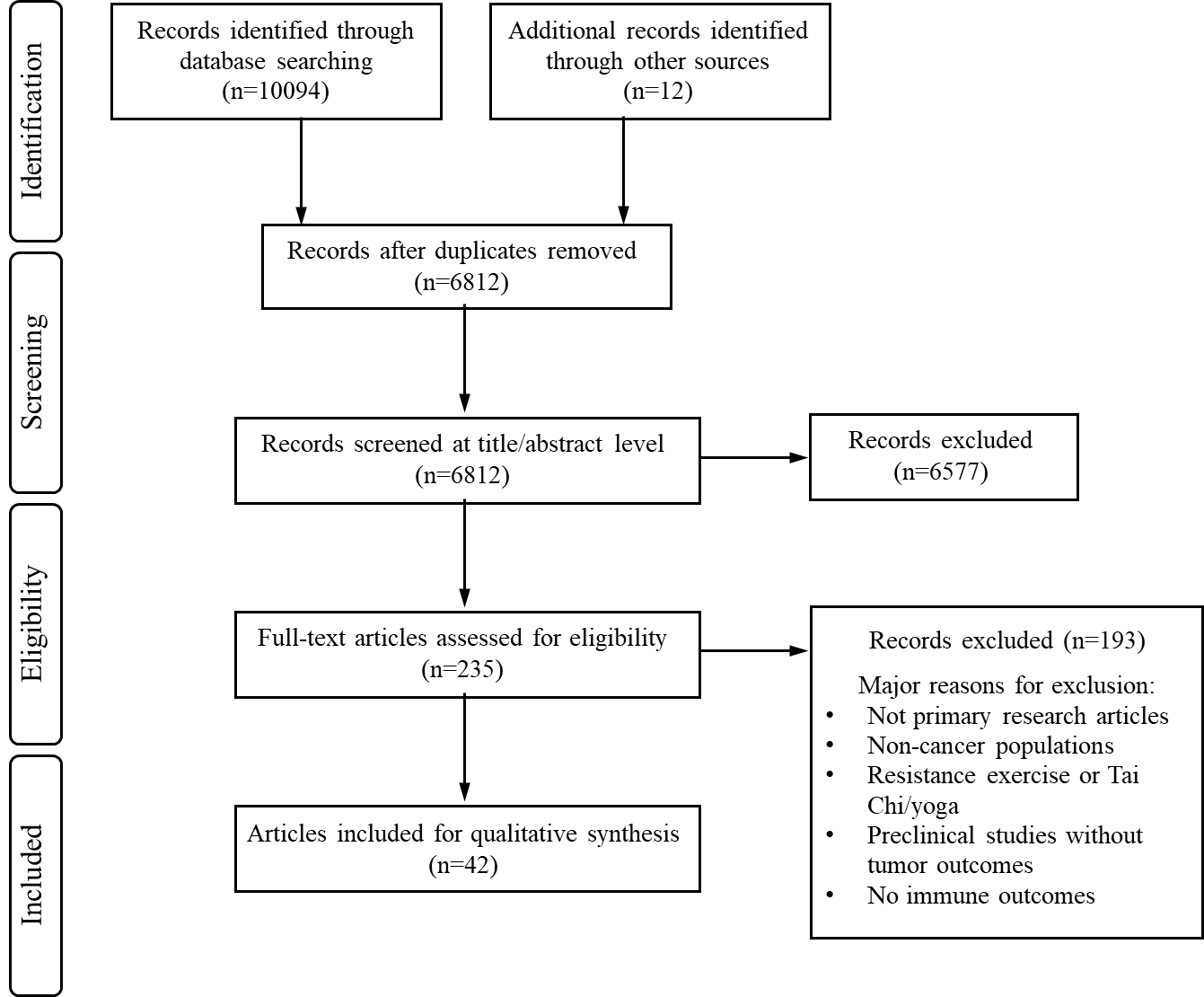


**Supplementary Figure 1.** PRISMA flow diagram of the study selection process.

Supplement: Supplementary file 4 [file Data_Sheet_1.docx]
